# Isolation, Economic Precarity, and Previous Mental Health Issues as Predictors of PTSD Status in Females Living in Fort McMurray During COVID-19

**DOI:** 10.3389/fpsyt.2022.837713

**Published:** 2022-03-18

**Authors:** Hannah Pazderka, Reham Shalaby, Ejemai Eboreime, Wanying Mao, Gloria Obuobi-Donkor, Belinda Agyapong, Folajinmi Oluwasina, Medard Kofi Adu, Ernest Owusu, Adegboyega Sapara, Vincent I. O. Agyapong

**Affiliations:** ^1^Department of Psychiatry, University of Alberta, Edmonton, AB, Canada; ^2^Be Brave Ranch, Centre for Treatment of Child Sexual Abuse, Edmonton, AB, Canada; ^3^Global Psychological E-Health Foundation, Edmonton, AB, Canada; ^4^Department of Psychiatry, Dalhousie University, Halifax, NS, Canada

**Keywords:** trauma, collective trauma, precarity, isolation, COVID-19, PTSD, resilience, retraumatization

## Abstract

**Objectives:**

The COVID-19 pandemic represents an instance of collective trauma across the globe; as such, it is unique to our lifetimes. COVID-19 has made clear systemic disparities in terms of access to healthcare and economic precarity. Our objective was to examine the mental health repercussions of COVID-19 on adult females living in Fort McMurray, Canada in light of their unique circumstances and challenges.

**Method:**

To investigate this issue, we analyzed responses gathered from an anonymous cross-section of online survey questionnaire responses gathered from females living in the Fort McMurray area (*n* = 159) during the COVID-19 pandemic (April 24–June 2, 2021). This included relevant demographic, mental health history, and post-traumatic stress disorder (PTSD), as well as COVID-19 data. Chi-squared analysis was used to determine outcome relevance, and binary logistic regression was employed to generate a model of susceptibility to PTSD.

**Results:**

159 females completed the survey. The prevalence of putative PTSD in our sample was 40.8%. A regression analysis revealed 4 variables with significant, unique contributions to PTSD. These were: a *diagnosis of depression*; a *diagnosis of anxiety*; *job loss due to COVID-19*; and *lack of support from family and friends*. Specifically, women with a previous diagnosis of either depression or anxiety were ~4–5 times more likely to present with PTSD symptomatology in the wake of COVID-19 (OR = 3.846; 95% CI: 1.13–13.13 for depression; OR = 5.190; 95% CI: 1.42–19.00 for anxiety). Women who reported having lost their jobs as a result of the pandemic were ~5 times more likely to show evidence of probable PTSD (OR = 5.182; 95% CI: 1.08–24.85). Receiving inadequate support from family and friends made the individual approximately four times as likely to develop probable PTSD (OR = 4.258; 95% CI: 1.24–14.65), while controlling for the other variables in the regression model.

**Conclusions:**

Overall, these results support our hypothesis that volatility in factors such as social support, economic stability, and mental health work together to increase the probability of women developing PTSD in response to a collective trauma such as COVID-19.

## Introduction

In March 2020, regions across the globe were impacted by the spread of the SARS CoV-2 virus (COVID-19). COVID-19 has had a large and devastating impact on mental health, in part due to increased social isolation (lockdowns, social distancing, and potential negative effects on relationships), economic ramifications (industries which could not withstand lockdown, job losses in some high contact sectors), and effects of the illness itself. According to one meta-analysis, the overall prevalence of stress in the general population due to COVID-19 approaches 30% (1). Negative mental health effects have been observed in groups as disparate as university students (2) and seniors (3, 4).

It is possible that some of these effects unevenly impact women. RBC economics reports^1^ that females were disproportionately affected by job loss, with women making up the majority of labor force exits, particularly in the service sector (5). Moreover, while men categorized themselves as “unemployed” (i.e., actively looking for work), many females changed their status to “out of the labor force”. These decisions appear to have been driven by the presumed need for females to stay home and take care of family members. Yet, the pandemic also resulted in increases in household conflict and gender-based violence (6), with women being unsure of how to access support services due to COVID-19, and related concerns about overburdening the system. Accordingly, higher rates of anxiety were observed in women during the pandemic (4).

We were interested in the impact of COVID-19 on rates of likely post-traumatic stress disorder (PTSD) among female residents of Fort McMurray, Alberta, a remote northern town. Fort McMurray has a large (almost 20% of residents) “shadow population” (7), most living in temporary accommodations and labor camps (8). Until the recent price collapse, the economic driver for the townsite has historically been the nearby oil sands. Accordingly, the town population is skewed toward being young (over 47% aged 20 to 44) (9) and male,^2^ (roughly 54.0% of the population, compared to 49.1% nationally) (10). A 2013 crime statistics report (11) notes that the region experiences relatively high rates of cocaine distribution and impaired driving. Fort McMurray also collects a very large proportion of video lottery terminal revenue in the province, second only to neighboring High Level (12). Overall, the town has a reputation of being somewhat patriarchal, catering mainly toward young males working in the resource industry (13). One magazine article went so far as to argue that Fort McMurray “has become synonymous with crime, an explosion in prostitution and the tough, young, bored single men with too much money and little to do” (14). Perhaps not surprisingly, the impact of COVID-19 in the surrounding region higher than average, with rates several times the provincial average (2045.2 vs. 574.4 per 100,000).^3^

The situation for females in Fort McMurray is decidedly different. It has been argued that, “[b]eliefs and visions of women's identities in resource communities and industries are especially prone to extreme polarization” (13), limiting their opportunities. In situations where women's options are already marginalized, it is reasonable to suggest that pandemic restrictions might have narrowed them still further (15). This may make females a vulnerable population in terms of dealing with COVID-19. For instance, women primarily dependent on spousal income may have simply left their jobs due to the pandemic while single women could not. Lockdowns may have also affected women disproportionately from involvement in social and community activities. Thus, power asymmetries, borne of pre-existing social structures, generate “unequal exposure to risk, making some groups of people… more prone to hazards” (16), including mental health risks associated with collective trauma. We speculate that females living in Fort McMurray may be at greater risk for post-traumatic consequences of COVID-19.

It has also been suggested that social support prior to a disaster appears to affect one's mental health adjustment to a collective trauma (17). Effects have been demonstrated in terms of both depressive symptomatology (18) and psychological distress (19)—although the latter showed no increase in post-traumatic stress. Nonetheless, a meta-analysis found “lack of support” to be one of the top three predictors of PTSD risk (20). We theorized that individuals who were in a relationship at the time of COVID-19 would fare better in terms of PTSD symptomatology than those living alone. Since previous research in Fort McMurray found that female residents had higher PTSD rates in response to the wildfire than men (21), and because reviews have suggested that, generally, women are more likely than men to experience PTSD in the wake of disasters (20, 22, 23), we focused our analysis exclusively on females. Both the living situations and the stressors experienced by women in Fort McMurray are likely different from those of local men.

Thus, this paper has two related objectives. The first is to look at the impact of relationship status of females on variables that appear to be important for dealing with adversity. The second is to examine potential predictors of PTSD in response to the COVID-19 pandemic.

## Methods

### Survey Details

Questionnaire responses were gathered via an online survey from a cross-section of Fort McMurray residents during the COVID-19 pandemic (April–June 2021). Data represent all responses received from respondents who identified as female, during this time period. Responses were self-reported, and participation was anonymous, via the secure, browser-based REDCap program (24). The questionnaire was comprised of demographic questions, self-reported history of common mental health disorders including PTSD [the PCL-C (25)], and questions pertaining to COVID-19. Consent was implied via survey completion. The study received approval from the University of Alberta Research Ethics Board (Pro00066054).

### Statistical Analysis

Data were analyzed using SPSS v25 (26). Descriptive statistics were analyzed for demographic, clinical, and COVID-19 related variables, based on relationship status. Chi-squared/Fisher exact analysis then examined the variables in relation to putative PTSD status. Binary logistic regression analysis identified potentially important predictors of PTSD, excluding strongly intercorrelated predictor variables (Spearman's correlation coefficients ≥0.7). The model included all significant (*p* ≤ 0.05) or nearly significant (*p* < 0.10) variables identified in the univariate analysis. Odds ratios and confidence intervals were calculated, to determine the association between each of the predictor variables and self-reported levels of PTSD. Missing data were not imputed; data represent all responses obtained.

### Sample Size Estimation

With a population of 111,687 as of the 2018 municipal census^4^ a 95% confidence interval, and a ± 3% margin of error, the sample size needed for prevalence rate estimates for PTSD would be 1058.

## Results

Of the 249 residents who accessed the online survey, 186 ultimately completed it, yielding a response rate of 74.7%. Of those 186, 85% (159) were females. Our analysis is limited to this subgroup of respondents.

### Demographics and Mental Health Status

As we were interested in how relationship status might affect life circumstances in light of the pandemic (i.e., the added stress of having to rely solely on one's own income; lack of emotional support), demographic variables were examined separately as a function of relationship status. Chi-squared tests were used to assess whether there were group differences between the different relationship status groups.

In the overall group, 115 (72.3%) were in a relationship, while 44 (27.7%) were not. Females who were not in a relationship were approximately the same age (54.5% over age 40 vs. 48.7% ≤ 40, ns; all results presented as “not in a relationship” vs. “married/co-habiting”, respectively) and equally likely to be employed (95.5% vs. 94.8%, *ns*). Interestingly, although the numbers were too small to draw any conclusions, nearly twice as many single females were employed in the oilsands (3.7%) as their married/co-habiting comparison group (7.1%). Jobs in this sector are far more likely to be contract positions, compared to some of the other sectors in the survey (e.g., government; school boards; healthcare), potentially suggesting higher precarity. Unmarried females were statistically twice as likely (36.4%) to be renting their accommodations as married women (13.9%; χ^2^ = 9.98, *p* < 0.01), again possibly linked to decreased security in terms of living circumstances.

Women not in a relationship showed a tendency toward a higher probability of some previous mental health diagnosis (63.6% vs. 48.7%; χ^2^ = 2.85, *p* < 0.10), and were almost twice as likely to report having had a previous mental health diagnosis of depression (45.5% vs. 27.8%; χ^2^ = 4.49, *p* < 0.05). In terms of current use of psychotropic medications, single women were more likely to have a prescription than were married/co-habiting women (50.0% vs. 30.4%; χ^2^ = 5.30, *p* < 0.05). Specifically, women not in a relationship were almost twice as likely to be on antidepressants as were those in a relationship (50.0% vs. 26.1%; χ^2^ = 8.27, *p* < 0.01). There was also a trend toward higher use of mood stabilizers (11.4% vs. 3.5%; χ^2^ = 3.71, *p* < 0.10), although numbers were low and should be viewed with caution. Similarly, women who were single were slightly more likely to have received mental health counselling in the past year (45.5% vs. 37.4%, *ns*) and to report wanting counselling (61.4% vs. 53.9%, *ns*), although neither of these differences was significant. Taken together, these data suggest that women not in a relationship were at higher risk for negative mental health effects as a result of COVID-19.

While one's relationship status might have some impact on dealing with difficult circumstances, it is also plausible that it might make some difference in terms of one's level of exposure to COVID-19 and its sequelae. In fact, we found no significant difference between the two groups with regard to these variables. Thus, the rest of this section reports on this group as a whole.

The vast majority (95.3%) of respondents reported being fearful about contracting the virus, and approximately the same proportion (97.3%) reported feeling fearful about friends or family contracting it. Just over two-thirds of respondents reported that their close friends or family had actually gotten sick with COVID-19 (69.8%). Similarly, almost two-thirds (61.7%) of individuals in the two groups reported having to self-isolate or quarantine due to symptoms or suspected contact with an affected individual. However, the vast majority did not lose their job due to COVID-19 (87.3%).

In terms of exposure to pandemic-related media imagery, 43.3% said they saw images depicting sick or dying people daily, and approximately the same number (42.7%) said they saw it occasionally, while only 14.0% reported never seeing this type of imagery.

The support individuals reported receiving was substantial, with approximately three-quarters (74.5%) of respondents saying they received moderate to high levels of family support. That said, reported levels of support by the federal government were quite a bit lower, with only 29.5% of respondents having received financial support from the Government of Canada. Provincial financial relief was about the same level, reported by 26.5% of respondents. Interestingly, these results stand in stark contrast to support provided by the individuals' employer, with almost three-quarters (73%) reporting moderate to high support from these local sources. These results are presented in Table 1.

**Table 1:** Demographic profile, clinical characteristics, and responses to COVID-19 of female respondents in Fort McMurray, as a function of relationship status.

**Variable**	**In a relationship *n* (%)**	**Not in a relationship *n* (%)**	**Total *n* (%)**	**Chi square/Fisher Exact**	***p*-value**	**Effect size (Cramer's V)**
**DEMOGRAPHIC CHARACTERISTICS**						
**Age**						
≤ 40y >40y	59 (51.3) 56 (48.7)	20 (45.5) 24 (54.5)	79 (49.7) 80 (50.3)	0.436	0.509	0.052
**Employment status**						
Employed Unemployed	109 (94.8) 6 (5.2)	42 (95.5) 2 (4.5)	151 (95.0) 8 (5.0)	0.30	0.862	0.014
**Housing status**						
Own home Renting	99 (86.1) 16 (13.9)	28 (63.6) 16 (36.4)	127 (79.9) 32 (20.1)	9.978	**0.002**	0.251
**CLINICAL CHARACTERISTICS**						
**Past mental health diagnosis (any)**						
No Yes	59 (51.3) 56 (48.7)	16 (36.4) 28 (63.6)	75 (47.2) 84 (52.8)	2.851	0.091	0.134
**Past mental health diagnosis (specific)**						
Depression Bipolar Disorder Anxiety Schizophrenia Personality Disorder Other	32 (27.8) 3 (2.6) 47 (40.9) 0 (0.0) 0 (0.0) 9 (7.8)	20 (45.5) 2 (4.5) 22 (50.0) 0 (0.0) 2 (4.5) 3 (6.8)	52 (32.7) 5 (3.1) 69 (43.4) 0 (0.0) 2 (1.3) 12 (7.5)	4.494 ^**^ 1.080 – ^**^0.046	**0.034** **0.617** 0.372 −0.075 0.830	0.168 0.050 0.082 −0.182 0.017
**Presently taking psychotropic medication (any)**						
No Yes	80 (69.6) 35 (30.4)	22 (50.0) 22 (50.0)	102 (64.2) 57 (35.8)	5.297	**0.021**	0.183
**Presently taking psychotropic medication (specific)**						
Antidepressants Antipsychotics Benzodiazepines Mood stabilizers Sedative hypnotics Other	30 (26.1) 1 (0.9) 3 (2.6) 4 (3.5) 10 (8.7) 2 (1.7)	22 (50.0) 0 (0.0) 1 (2.3) 5 (11.4) 7 (15.9) 0 (0.0)	52 (32.7) 1 (0.6) 4 (2.5) 9 (5.7) 17 (10.7) 2 (1.3)	8.269 ^**^ ^**^ 3.706 1.734 ^**^	**0.004** 1.00 1.00 0.054 0.188 1.00	0.228 0.049 0.010 0.153 0.104 0.070
**Has received MH counselling in past year**	43 (37.4)	20 (45.5)	63 (39.6)	0.865	0.352	0.074
**Would like to receive MH counselling**	62 (53.9)	27 (61.4)	89 (56.0)	0.717	0.397	0.067
**COVID-19 VARIABLES**						
Reports having been fearful about contracting the coronavirus	104 (94.5)	39 (97.5)	143 (95.3)	0.576	0.448	0.062
Reports having been fearful about close friends/family members contracting the coronavirus	107 (97.3)	39 (97.5)	146 (97.3)	^**^	0.99	0.006
Reports having close friends or family members who have been sick from the coronavirus disease	80 (73.4)	24 (60.0)	104 (69.8)	2.490	0.115	0.129
Had to self-isolate or self-quarantine due to symptoms, recent travel, or contact with someone who may have COVID-19?	69 (63.3)	23 (57.5)	92 (61.7)	0.417	0.518	0.053
**Lost job due to the COVID-19 pandemic**						
No Yes	97 (88.2) 13 (11.8)	34 (85.0) 6 (15.0)	131 (87.3) 19 (12.7)	0.268	0.604	0.042
**Frequency of viewing television images of sick and dead people caused by coronavirus**						
Daily Less than daily Never	49 (44.5) 48 (43.6) 13 (11.8)	16 (40.0) 16 (40.0) 8 (20.0)	65 (43.3) 64 (42.7) 21 (14.0)	1.633	0.442	0.104
**Frequency of reading newspaper and internet articles related to the pandemic**						
Daily Less than daily Never	62 (56.4) 45 (40.9) 3 (2.7)	25 (62.5) 15 (37.5) 0 (0.0)	87 (58.0) 60 (40.0) 3 (2.0)	1.337	0.505	0.095
**Received sufficient support from family and friends for COVID-19 pandemic**						
Some-to-high level support Limited or no support	82 (75.2) 27 (24.8)	29 (72.5) 11 (27.5)	111 (74.5) 38 (25.5)	0.115	0.735	0.028
**Received sufficient support from Government of Canada for COVID-19 pandemic**						
Some-to-high level support Limited or no support	33 (30.8) 74 (69.2)	10 (25.6) 29 (74.4)	43 (29.5) 103 (70.5)	0.372	0.542	0.050
**Received sufficient support from Government of Alberta for COVID-19 pandemic**						
Some-to-high level support Limited or no support	27 (28.7) 67 (71.3)	8 (21.1) 30 (78.9)	35 (26.5) 97 (73.5)	0.817	0.366	0.079
**Received sufficient support from the employer for COVID-19 pandemic**						
Some-to-high level support Limited or no support	81 (75.0) 27 (25.0)	27 (67.5) 13 (32.5)	108 (73.0) 40 (27.0)	0.833	0.362	0.075

** *Indicates use of Fisher exact statistic. Bold values indicate signif./near-signif. effects*.

Overall, 40.8% of the respondents to the survey showed evidence of a likely PTSD diagnosis. A univariate analysis examined the relationship between respondents' demographic profile, mental health status, and COVID-19 experience, with probable PTSD. Overall, 16 variables were significant or near-significant predictors of PTSD, including: *employment status, relationship status, previous mental health diagnosis* (any, depression, anxiety), *current medication status* (any, antidepressants, benzodiazepines, sedative-hypnotics), *mental health counselling in the past year, desire to receive mental health counselling*, and a number of COVID-19 variables, including being *fearful about contracting Covid, pandemic-related job loss, family support, government support*, and *employer support*. These results are presented in Table 2.

**Table 2:** Chi square test of association between demographic variables, mental health status, COVID-19 characteristics, and probable PTSD.

**Variable**	**Not likely PTSD**	**Likely PTSD**	**Chi square/ Fisher Exact**	***p*- value**	**Effect size (Cramer's V)**
**DEMOGRAPHIC CHARACTERISTICS**					
**Age categories**					
≤ 40y >40y	40 (58.8) 44 (59.5)	28 (41.2) 30 (40.5)	0.006	0.939	0.006
**Employment status**					
Employed Unemployed	84 (61.8) 0 (0.0)	52 (38.2) 6 (100.0)	^**^	**0.004**	0.253
**Relationship status**					
In a relationship Not in a relationship	66 (63.5) 18 (47.4)	38 (36.5) 20 (52.6)	2.98	**0.084**	0.145
**Housing status**					
Own home Renting	67 (58.8 17 (60.7)	47 (41.2) 11 (39.3)	0.035	0.851	0.016
**CLINICAL CHARACTERISTICS**					
**Past mental health diagnosis: Any**					
No Yes	52 (77.6) 32 (42.7)	15 (22.4) 43 (57.3)	17.885	**0.000**	0.335
**Past mental health diagnosis: Depression**					
No Yes	69 (72.6) 15 (31.9)	26 (27.4) 32 (68.1)	21.575	**0.000**	0.390
**Past mental health diagnosis: Bipolar Disorder**					
No Yes	82 (59.4) 2 (50.0)	56 (40.6) 2 (50.0)	^**^	1.00	0.032
**Past mental health diagnosis: Anxiety**					
No Yes	61 (75.3) 23 (37.7)	20 (24.7) 38 (62.3)	20.36	**0.000**	0.379
**Past mental health diagnosis: Personality Disorder**					
No Yes	83 (58.9) 1 (100.0)	58 (41.1) 0 (0.0)	^**^	1.00	0.070
**Current medications: Any**					
No Yes	61 (67.0) 23 (45.1)	30 (33.0) 28 (54.9)	6.508	**0.011**	0.214
**Current medications: Antidepressants**					
No Yes	64 (66.7) 20 (43.5)	32 (33.3) 26 (56.5)	6.921	**0.011**	0.221
**Current medications: Benzodiazepines**					
No Yes	84 (60.9) 0 (0.0)	54 (39.1) 4 (100.0)	^**^	**0.026**	0.205
**Current medications: Mood stabilizers**					
No Yes	81 (60.0) 3 (42.9)	54 (40.0 4 (57.1)	^**^	0.444	0.075
**Current medications: Sedative-hypnotics**					
No Yes	81 (63.8) 3 (20.0)	46 (36.2) 12 (80.0)	10.642	**0.002**	0.274
**Current medications: Other psychotropic**					
No Yes	82 (58.6) 2 (100.0)	58 (41.4) 0 (0.0)	^**^	0.513	0.099
**Received mental health counselling in the past year**					
No Yes	63 (71.6) 21 (38.9)	25 (28.4) 33 (61.1)	14.81	**0.000**	0.323
**Reports wanting to receive mental health counselling**					
No Yes	51 (81.0) 33 (41.8)	12 (19.0) 46 (58.2)	22.268	**0.000**	0.396
**COVID-19 VARIABLES**					
**Reports having been fearful about contracting the coronavirus**					
No Yes	5 (71.4) 79 (58.5)	2 (28.6) 56 (41.5)	^**^	**0.070**	0.057
**Reports having been fearful about close friends/family members contracting the coronavirus**					
No Yes	4 (100.) 80 (58.0)	0 (0.0) 58 (42.0)	^**^	0.145	0.141
**Reports having close friends or family members who have been sick from the coronavirus disease**					
No Yes	24 (54.5) 60 (61.2)	20 (45.5) 38 (38.3)	0.561	0.454	0.063
**Had to self-isolate or self-quarantine due to symptoms, recent travel, or contact with someone who may have COVID-19?**					
No Yes	31 (58.5) 53 (59.6)	31 (58.5) 53 (59.6)	0.015	0.901	0.010
**Lost job due to the COVID-19 pandemic**					
No Yes	79 (64.2) 5 (26.3)	44 (35.8) 14 (73.7)	9.790	**0.002**	0.263
**Frequency of viewing television images of sick and dead people caused by coronavirus**					
Daily Less than daily Never	34 (56.7) 41 (64.1) 9 (50.0)	26 (43.3) 23 (35.9) 9 (50.0)	1.416	0.493	0.100
**Frequency of reading newspaper and internet articles related to the pandemic**					
Daily Less than daily Never	46 (56.1) 36 (63.2) 2 (66.7)	36 (43.9) 21 (36.8) 1 (33.3)	0.765	0.682	0.073
**Received sufficient support from family and friends for COVID-19 pandemic**					
Some-to-high level support Limited or no support	71 (66.4) 12 (35.3)	36 (33.6) 22 (64.7)	10.28	0.0**01**	0.270
**Received sufficient support from Government of Canada for COVID-19 pandemic**					
Some-to-high level support Limited or no support	28 (70.0) 53 (53.5)	12 (30.0) 46 (46.5)	3.176	0.0**75**	0.151
**Received sufficient support from Government of Alberta for COVID-19 pandemic**					
Some-to-high level support Limited or no support	24 (75.0) 56 (58.9)	8 (25.0) 39 (41.1)	2.646	0.104	0.144
**Received sufficient support from the employer for COVID-19 pandemic**					
Some-to-high level support Limited or no support	70 (66.0) 13 (37.1)	36 (34.0) 22 (62.9)	9.073	**0.003**	0.254

** *Indicates use of Fisher exact statistic. Bold values indicate signif./near-signif. effects*.

All variables that demonstrated asymptotic significance were then tested as potential predictors in the logistic regression model. Two of these variables were ultimately removed, as high correlations with other variables would have meant they were duplicating other data in the model. These were: *history of any mental health diagnosis* (which showed high correlations with both a depression or anxiety diagnosis, as well as with medication use), and *antidepressant use* (which correlated highly with mental health status and medication use more generally). Two other variables—*current employment status* and *benzodiazepine use*—were also dropped from the model due to low overall *n*'s in some cells. Thus, the model ultimately retained 12/16 potential chi-squared predictors.

The overall model was statistically significant; χ^2^ (d*f* = 12; *n* = 137) = 66.12, *p* < 0.001, suggesting it could distinguish between respondents who did or did not go on to develop probable PTSD during the pandemic. Variables in the model accounted for between 38.3% (Cox and Snell *R*^2^) and 51.5% (Nagelkerke *R*^2^) of the overall variance. The model correctly classified 81.0% of all cases (82.3% of individuals without PTSD, and 79.3% of those with likely PTSD). Results of the multivariate logistic regression model used to predict likely PTSD are presented in Table 3.

**Table 3:** Logistic regression model for respondents' likelihood to present with probable PTSD.

**Characteristics**	**Coefficient**	**S.E**.	**Wald statistic**	***p*-value**	**Odds Ratio**	**95% C.I. for Odds Ratio**	
						**Lower**	**Upper**
Relationship status	0.468	0.514	0.829	0.362	1.597	0.583	4.376
Depression diagnosis	1.347	0.626	4.624	**0.032**	3.846	1.127	13.129
Anxiety diagnosis	1.647	0.662	6.186	**0.013**	5.190	1.418	18.999
Not on any medication for mental health concerns	−1.449	0.783	3.423	0.064	0.235	0.051	1.090
Medication (sedative-hypnotics)	1.659	0.902	3.383	0.066	5.256	0.897	30.799
Counselling past year	0.900	0.624	2.081	0.149	2.459	0.724	8.351
Would like to receive counselling	0.605	0.578	1.096	0.295	1.832	0.590	5.689
Fearful about the COVID-19 pandemic	0.072	1.198	0.004	0.952	1.074	0.103	11.238
Job loss due to COVID-19 pandemic	1.645	0.800	4.230	**0.040**	5.182	1.081	24.849
Limited or no support from family and friends for COVID-19 pandemic	1.449	0.630	5.284	**0.022**	4.258	1.238	14.649
Limited or no support from Government of Canada for COVID-19 pandemic	0.275	0.587	0.220	0.639	1.317	0.417	4.159
Limited or no support from employer for COVID-19 pandemic	0.793	0.558	2.023	0.155	2.210	0.741	6.591
Constant	−3.075	1.356	5.140	0.023	0.046		

*Bold values indicate signif./near-signif. effects*.

Four variables showed significant predictive power in terms of identifying probable PTSD in the model, after controlling for other potential causes. These were: *diagnosis of depression, diagnosis of anxiety, job loss due to COVID-19*, and *lack of family support*. Respondents who had a diagnosis of depression were almost four times as likely to develop PTSD symptoms as those without (OR = 3.846; 95% CI: 1.13–13.13). Similarly, respondents with a prior diagnosis of anxiety were five times as likely (OR = 5.190; 95% CI: 1.42–19.00). Losing one's job as a result of COVID-19 made them approximately five times more likely to develop this diagnosis (OR = 5.182; 95% CI: 1.08–24.85). Finally, receiving little or no family support made one approximately four times as likely to develop probable PTSD (OR = 4.258; 95% CI: 1.24–14.65). It is worth noting that relationship status did not make a significant, unique contribution to the model, counter to our prediction.

## Discussion

Our study focused on the prevalence and the predictors of PTSD arising from COVID-19 among females in Fort McMurray. Over 40% of respondents showed evidence of likely PTSD. This number is quite high compared to rates reported in a systematic review of other natural disasters (27). It is, however, comparable to our previous research, where Fort McMurray adolescents showed rates exceeding 37%, even 4.5 years after the wildfire (28). It is worth considering whether these rates are elevated due to the large amount of previous trauma faced by this town: Inhabitants confronted the devastating wildfire in 2016, were hit by the oil price crash, and faced flooding in 2020, along with the onset of COVID-19. This means that their population faced many consecutive traumas. As one resident put it in a news report^5^ following the flood, “That odd feeling I felt 4 years ago when we had to evacuate because of the wildfire is that same old feeling again… [Except t]his time, there's that extra worry because we have the pandemic” (29). It is plausible that the PTSD seen in our sample following the pandemic really reflects the cumulative effects of multiple traumas. This is in line with previous research suggesting that additional life stressors—particularly those severe enough to result in PTSD (17) and those that could be classified as continuous life stress (30)—put people at increased risk for developing PTSD following a disaster. A number of factors were shown to be predictive of negative mental health in response to the pandemic for women in Fort McMurray. Our finding that a prior diagnosis of either depression or anxiety significantly predicted developing PTSD symptoms supports the idea that existing mental health difficulties put one at greater risk in the event of a collective trauma. To the extent that depression and anxiety may reflect responses to prior trauma (e.g., adverse childhood events), it would seem that these results support the idea that prior trauma increases reactivity to and potential harm of new trauma.

Interestingly, media exposure was not found to make a difference in terms of whether one was likely to develop symptoms of PTSD. In fact, individuals who were in the Not Likely PTSD group reported more daily exposure to COVID-19 news and imagery than those in the Likely group. This is somewhat surprising, given predictions of negative effects associated with our 24-hour news cycle (31). Moreover, being fearful about the pandemic was also not a good predictor of who went on to develop PTSD. Both of these findings point to the conclusion that, as Neria and Sullivan (30) suggest, “longer-term PTSD to [media] exposure may have more to do with the preexisting vulnerability than with the indirect exposure severity *per se*” (p. 3). In other words, neither exposure to negative information, nor anxiety about the stressor itself, appear to be good predictors of who goes on to develop PTSD.

The finding that job loss was associated with developing probable PTSD fits with our hypothesis that economic precarity puts one at risk for mental health issues. In some sense this is unsurprising, as unemployment has been shown to negatively affect mental health (32, 33). However, this finding may be more specific to COVID-19, as the pandemic directly affected employment for some individuals but not others. McKee-Ryan and co-workers (32) identified four factors as mediating the relationship between unemployment and mental health—work-role centrality, coping resources, cognitive appraisals, and coping strategies—all of which could potentially have been impacted by COVID-19. That said, it is noteworthy that neither government nor employer support had a measurable effect on who showed probable PTSD. It might be that, while Canada provided comparatively generous financial support (the Canadian Emergency Response Benefit, or CERB) to those negatively impacted economically by COVID-19, it may still have been insufficient to meet the needs of individuals living in Fort McMurray, which has unusually high rates of inflation and housing costs. Similarly, job sharing agreements, which were instituted in many work environments with the advent of COVID-19 (34), may not have had general applicability to positions in work camps, many of which were contract positions. In other words, these forms of government support may have had limited applicability to this specific population, or been insufficient to meet their needs. Finally, there may have been difference in terms of how job loss was experienced in different sectors. It would appear that the effects of economic instability are not necessarily symmetrical, with consequences of job loss having worse mental health effects than positive impacts of employment support.

The finding that lack of social support was predictive of probable PTSD in women fits with the idea that social isolation is associated with greater potential harm in the context of collective trauma. A recent literature review suggested that high rates of support prior to natural disasters appear to mitigate psychological trauma in the aftermath of a crisis (35). This is interesting in terms of the lack of effects of governmental and employer support discussed above; it would appear that supports might need to be more personal and less bureaucratic in order to have a positive impact. Having supportive loved ones = people to discuss potential fears, problem-solve solutions when problems arise, and express empathy when bad things happen—is undoubtedly advantageous. That said, contrary to our hypothesis [and the findings of other authors (36)], relationship status was *not* found to be predictive of developing post traumatic stress. It is possible that being in a relationship lacking emotional encouragement (e.g., downplaying the partner's fears about possible dangers due to skepticism, or not supporting the partner's coping strategies) may in fact be even more traumatizing than being alone. These types of negative “cognitive reappraisals” have been speculated as being key mechanisms in the link between social support and PTSD (37). In other words, being in a relationship cannot necessarily be equated to having social support.

Finally, we should note that relatively little work has been done to look at factors that increase vulnerability to PTSD in women as a result of COVID-19, specifically. While many papers note that being female is in itself a risk factor, they generally report on a wide variety of other risk factors [e.g., being a strong internet user (38), the specific classification of population to which the individual belongs (39), one's perception of racial discrimination (40) and one's perception of the pandemic as a crisis (36)]. Yet, women have been shown to have a different pattern in terms of the PTSD symptoms and sleep quality experienced during COVID-19 (41), suggesting potentially distinct physiological mechanisms at work. Supporting this idea, other authors suggest that the genders may even respond differently to peritraumatic stress (experienced during and immediately after the event) compared to posttraumatic stress (42). As we have argued here, there can be a number of factors that affect female vulnerability to stressors differentially than males—economic, social, and emotional. Females' different sociocultural milieu warrants consideration. We suggest that future studies should focus specifically on females and the issues they encountered with COVID-19 (and other mass traumas) specifically.

## Limitations

Our study has several limitations. The sample size is fairly small, and the individuals responding to this study constitute a self-selected group, and so do not represent a random sampling of all individuals, or even all females, in the region. In particular, the high rates of PTSD seen in this analysis likely reflect that people with intrusive and problematic symptoms will be more likely to seek resources online. Moreover, our overall low sample size resulted in fairly broad Confidence Intervals, which decrease the resultant certainty of the estimates we have put forth. Besides, as the sample size of this study was much less than the sample size estimates that we projected, the margin error of our prevalence estimates was ±7% rather than the ±3% which we projected initially.

In addition, due to the small overall population of Fort McMurray (<100,000), and the personal nature of the mental health data requested, precautions were taken to ensure that no combination of question responses could lead to identifiable data. This included limiting questionnaire items mainly to those concerned with mental health or COVID-19. Thus, we chose to exclude information regarding ethnicity and educational attainment, as well as some health items such as physical disease history, pregnancy status, and vaccination status. This means some potentially important effects were not examined. For example, one meta-analysis has reported that pregnancy history is associated with PTSD, with high-risk pregnancies resulting as in rates as high as 18%, either pre- or post-partum (43). This calls into the question some of our findings, as we cannot exclude the possibility that some of the respondents in our sample, who we know had gone through multiple traumas, might be pregnant. As another significant example, educational attainment has been linked to one's misperception of falsehoods regarding COVID-19 (44), leading to riskier behaviour and worse outcomes (45). Similarly, the decision to exclude benzodiazepine use as a predictor of PTSD due to low *n*'s is unfortunate, as some research attempting to use prophylactic benzodiazepines to prevent PTSD actually found a paradoxical increase in PTSD (46, 47) diagnosis. Future research on COVID-19 and other mass trauma incidents should seek to examine some of these factors and their impacts on PTSD more directly.

It should also be noted that PTSD categorizations were based upon questionnaire measures of mental health. These cannot replace clinical assessment. An individual may struggle with depression or anxiety, never discuss it formally with a doctor, and it may resolve itself. Thus, presumed rates of probable PTSD must be viewed as suggestive. More generally, self-report questionnaires can show validity problems, demand effects, and be prone to issues associated with recollection of facts and events.

This study is also subject to problems observed with cross-sectional analyses more generally. We do not have information on the ultimate effects of any predictor, simply the correlations simultaneously observed in the data, so cause-and-effect conclusions cannot be drawn. This study would benefit from follow-up research to determine whether the associations predicted by this analysis are confirmed longitudinally.

Finally, this study was limited to females, and results cannot be generalized beyond this subset of the population. For instance, it has been theorized that the pandemic exacerbated the precarious positions of women by reinforcing existing patriarchal structures while eroding female friendships given necessary measures such as lockdowns and social distancing (15). It is likely that variables such as social support and economic precarity due to collective trauma play a different role in terms of modeling potential mental health effects in men.

## Conclusion

This study focused on the prevalence of PTSD in female residents of Fort McMurray, Canada, in response to the COVID-19 pandemic. This community has experienced a series of negative events, economic as well as environmental, since the 2016 wildfire that made it the subject of national attention. This includes evacuation, relocation, rebuilding after the fire, the economic collapse of oil prices, flooding, and COVID-19. This string of events has led to a situation where life for residents never truly “got back to normal”. With climate crises becoming all the more common, our understanding of multiplicative effects of retraumatization will gain importance. This points to the necessity of broad governmental income security measures, such as a Basic Income, to help support communities in situations where multiple traumas occur. Our findings support the thesis that factors influencing this constant instability had negative mental health effects on the women of Fort McMurray. Instability, in terms of poor family support, economic precarity, and a history of mental health issues were all found to predict probable PTSD symptoms. Taken together, these results suggest that promoting interventions that contribute to a sense of stability is key to mental health recovery following collective trauma.

## Data Availability Statement

The raw data supporting the conclusions of this article will be made available by the authors, without undue reservation.

## Ethics Statement

The studies involving human participants were reviewed and approved by University of Alberta Ethics Research Board (Pro00066054). Written informed consent for participation was not required for this study in accordance with the national legislation and the institutional requirements.

## Author Contributions

The study was conceived and designed by VA. HP drafted the initial manuscript. RS conducted data analysis. All authors contributed to study design, review, and revision of the initial draft manuscript. The final draft was read and approved by all authors prior to submission.

## Funding

This study was funded by the Mental Health Foundation. Support with survey link distribution was received from the Fort McMurray Public and Catholic School Boards, Keyano College, as well as the Canadian Mental Health Association.

## Conflict of Interest

The authors declare that the research was conducted in the absence of any commercial or financial relationships that could be construed as a potential conflict of interest.

## Publisher's Note

All claims expressed in this article are solely those of the authors and do not necessarily represent those of their affiliated organizations, or those of the publisher, the editors and the reviewers. Any product that may be evaluated in this article, or claim that may be made by its manufacturer, is not guaranteed or endorsed by the publisher.

## Footnotes

^1^ https://thoughtleadership.rbc.com/canadian-women-continue-to-exit-the-labour-force/

^2^ https://www12.statcan.gc.ca/census-recensement/2016/dp-pd/prof/details/page.cfm?Lang=E&Geo1=POPC&Code1=0292&Geo2=PR&Code2=01&SearchText=Canada&SearchType=Begins&SearchPR=01&B1=All&TABID=1&type=0

^3^ https://www.alberta.ca/maps/covid-19-status-map.htm

^4^ https://www.rmwb.ca/en/permits-and-development/resources/Documents/Latest-Census-Report-2018.pdf

^5^ https://edmontonjournal.com/news/local-news/boil-water-advisory-issued-north-of-athabasca-river-evacuation-orders-issued-for-parts-of-downtown/
